# *Platycodon grandiflorus* Fermented Extracts Attenuate Endotoxin-Induced Acute Liver Injury in Mice

**DOI:** 10.3390/nu12092802

**Published:** 2020-09-13

**Authors:** So Ra Kim, Eun Jung Park, Theodomir Dusabimana, Jihyun Je, Kyuho Jeong, Seung Pil Yun, Hye Jung Kim, Kye Man Cho, Hwajin Kim, Sang Won Park

**Affiliations:** 1 Department of Pharmacology, Institute of Health Sciences, Gyeongsang National University College of Medicine, Jinju 52727, Korea; candylll@naver.com (S.R.K.); foreverpak1@nate.com (E.J.P.); odomy2020@gmail.com (T.D.); jeri1984@naver.com (J.J.); khjeong@gnu.ac.kr (K.J.); spyun@gnu.ac.kr (S.P.Y.); kimhyejung@gnu.ac.kr (H.J.K.); 2 Department of Convergence Medical Sciences, Institute of Health Sciences, Gyeongsang National University Graduate School, Jinju 52727, Korea; 3 Department of Food Science, Gyeongnam National University of Science and Technology, Jinju 52725, Korea; kmcho@gntech.ac.kr

**Keywords:** Acute liver injury, anti-inflammation, antioxidant, fermentation, nitric oxide, *Platycodon grandiflorus*

## Abstract

Endotoxin-induced acute liver injury is mediated by an excessive inflammatory response, hepatocellular oxidative stress, and apoptosis. Traditional medicinal plants have been used to treat various disorders. *Platycodon grandifloras* (PG) has been shown to be beneficial in relieving cough and asthma and to have anti-tumor, anti-inflammatory, anti-diabetic activities. The pharmacological action of PG is mainly due to saponins, flavonoids, phenolic, and other compounds. However, raw PG exhibits some side effects at high doses. Here, we extracted raw PG with varying fermentation methods and examined its anti-inflammatory effect and associated signaling kinases in Raw264.7 cells. Then, we investigated the effect of fermented black PG (FBPG) on endotoxin-induced liver injury. Mice were administered FBPG orally at 1 h before the lipopolysaccharide and D-galactosamine (LPS/GalN) injection and sacrificed after 5 h. Black PG (BPG) and FBPG showed a significant reduction in pro-inflammatory cytokines and extracellular nitric oxide (NO); p-38 and ERK signaling was involved in reducing inducible NO synthase in Raw264.7 cells. Consistently, FBPG attenuates LPS/GalN-induced liver injury; plasma ALT and AST, hepatic necrosis, pro-inflammatory cytokines, apoptosis, and lipid peroxidation were all reduced. In conclusion, PG extracts, particularly FBPG, play anti-inflammatory, antioxidant, and anti-apoptotic roles, alleviating endotoxin-induced acute liver injury. Processing raw PG into FBPG extract may be clinically useful by improving the pharmacologically active ingredients and reducing the required dosage.

## 1. Introduction

The liver is an essential organ involved in energy metabolism, protein synthesis, fat metabolism, glycemic control, and detoxification; thus, many patients with acute and chronic liver diseases suffer from multiple layers of serious complications. Acute liver failure (ALF) is defined by a rapid development of hepatic dysfunction (loss of 80–90% of liver) [1,2] within 8–28 days, complicated by coagulopathy and hepatic encephalopathy in patients without preexisting chronic liver disease [3]. ALF is caused by paracetamol (acetaminophen) overdose, excessive alcohol consumption, viral hepatitis, acute fatty liver of pregnancy, and idiopathic diseases [4]. Many researchers have developed experimental models, particularly using endotoxin-induced hepatotoxicity, to test the efficacy of new therapeutic agents and to understand better the ALF pathophysiology [5].

Animal models of liver injury include carbon tetrachloride-, galactosamine-, or alcohol-induced hepatotoxicity and choline deficiency; the addition of lipopolysaccharide (LPS), serving as cofactor, augments the hepatitis with severe hepatic congestion resulting in rapid death [6,7,8]. In these liver injury models, Kupffer cells are major sources of pro-inflammatory cytokines, such as tumor necrosis factor (TNF)-α, interleukin-6 (IL-6), and IL-18. Binding of LPS to the binding proteins (LBP) stimulates cluster of differentiation 14 (CD14) followed by activation of Toll-like receptor 4 (TLR-4), resulting in the translocation of nuclear factor kappa-light-chain-enhancer of activated B cells (NF-κB) and the production of pro-inflammatory cytokines [9]. Galactosamine (GalN) is a potent hepatotoxin that depletes uridine nucleotides (UTPs), which decreases RNA and protein synthesis, primarily in the liver; thus, GalN enhances sensitivity to elevated TNF-α levels [10].

TNF-α-mediated activation of neutrophils and apoptotic cell death associated with caspase activation are important pathological features of ALF, where the extent of hepatocyte death exceeds the hepatic regenerative and antioxidant capacities [5,11]. Hepatotoxicity during endotoxemia has been reported to be caused by neutrophil-derived oxidative stress via NADPH oxidase and is more susceptible to endotoxin in the glutathione peroxidase-deficient mice [12,13].

Recently, natural medicinal plant extracts have been shown to be beneficial for antioxidant, anti-inflammatory, and anti-apoptotic properties, which offer an attractive therapeutic strategy to reduce endotoxin-induced hepatotoxicity and further protect the liver from chronic damage. *Platycodon grandifloras* (PG) is a perennial herb belonging to the family *Campanulaceae* and has a long history of use as food and medicine in east Asian countries such as China, Japan, and Korea. The roots of PG are the most edible part of the plant containing high levels of nutrients, but tender seedlings are also consumed in salads or side dishes for their taste and color [14,15]. Previous work has shown therapeutic effects of PG on the respiratory system by relieving coughing, phlegm, and asthma symptoms. PG has also shown anti-inflammatory, antioxidant, anti-tumor, anti-diabetic, hepatoprotective, and other activities in various diseases [16]. PG has numerous bioactive components, including triterpenoid saponins, flavonoids, phenolic acids, polyacetylene, sterols, and amino acids [14,16].

Few studies have examined the effect of PG on endotoxin-induced liver injury as a model for pathogenesis of ALF and chronic inflammatory liver diseases. In this study, we processed raw PG with varying extraction and fermentation methods to improve pharmacologically active ingredients and prepared dried PG (DPG), black PG (BPG), and fermented BPG (FBPG). We first examined the anti-inflammatory effect in Raw264.7 cells in vitro, and then investigated the protective effect on an LPS/GalN-induced liver injury model in vivo.

## 2. Materials and Methods

### 2.1. Preparation of PG extracts

The roots of *Platycodon grandiflorus* were purchased from Jayeonae Biolab Co., Ltd. (Sancheong, Gyeongnam, Korea); PG was cultivated for over 2 years under controlled conditions. To prepare BPG, after washing three times, the roots were steamed at 100 °C for 60 min and aged at 75 °C in a closed container for 9 days. For additional fermentation to prepare FBPG, after adjusting the water content to 40–50%, 2% (*w*/*v*) of sucrose was added and sterilized at 121 °C for 30 min. Then, 5% (*v*/*v*) of 1:1 mixture of *Lactobacillus plantarum* (P1201) and *Lactobacillus brevis* (BMK184) was injected and fermented at 30 °C for 5 days. All samples were dried at 55 °C for 3 days, pulverized, and stored at −20 °C. For ethanol extraction, 100 mL of 50% ethanol was added to 10 g of each sample and extracted at 300 rpm for 12 h. The extracts were filtered at 0.45 μm and lyophilized at −70 °C. The extract preparation process was standardized as previously described [17,18].

### 2.2. High-Performance Liquid Chromatography (HPLC)

The analysis was performed on an Agilent 1200 Infinity series (Agilent technologies, Santa Clara, CA, United States). Samples were separated on a TSKgel ODS-100Z analytical column (Merck KGaA, Darmstadt, Germany). The phenolic acid and flavonoid analysis used the mobile phase containing 2% glacial acetic acid in water (Solution A) and 2% glacial acetic acid in acetonitrile (Solution B). The ratio of solution based on B was 15%, 5%, 15%, 5%, 10%, 50%, 50%, 60%, 80%, and 90% at 10, 15, 20, 25, 30, 35, 40, 45, 55, and 60 min, respectively. The injection volume was 20 μL, and the flow rate was 1.0 mL/min at 30 °C. The detection wavelength was 280 and 270 nm for phenolic acids and flavonoids, respectively. The triterpenoide analysis used the mobile phase containing HPLC water (Solution A) and acetonitrile (Solution B). The ratio of solution based on B was 19%, 20%, 23%, 30%, 35%, 70%, and 90% at 0, 15, 40, 42, 75, 80, and 90 min, respectively. The injection volume was 10 μL, the flow rate was 1.0 mL/min at 30 °C, and the detection wavelength was 203 nm.

### 2.3. Animals

Male C57BL/6 mice (8-week old) were purchased from KOATECH Co. (Pyeongtaek, South Korea) and maintained in a group of 4–5 mice per cage, in the animal facility at Gyeongsang National University. All animal experiments were approved by the Institutional Board of Research at Gyeongsang University (GNU-200820-M0052) and were performed in accordance with the National Institutes of Health guidelines for laboratory animal care. Mice were housed in the Mouse Blue Vent isocage system (polyphenylsulfone type) from Three-Shine Inc. (Daejeon, South Korea), maintained at temperatures of 22–24 °C with 40–60% humidity. Mice were housed under an alternating 12-h light/dark cycle and provided with sterilized water and standard diet (2018S) from Harlan Laboratories (Indianapolis, IN, USA). Mice were fasted for 18 h before experiments.

### 2.4. An Acute Liver Injury Model and Sample Collection

C57BL/6 mice were randomly divided into four groups: sham mice (*n* = 5) and mice treated with vehicle (water) or FBPG at 10 or 100 mg/kg 1 h prior to LPS/GalN injection (*n* = 8, each). FBPG was administered orally at 1 h before the intraperitoneal injection of LPS (L4130, Sigma-Aldrich) and GalN (G0500, Sigma-Aldrich) at 10 μg/kg and 700 mg/kg, respectively, as previously reported [19,20,21]. Then, the mice were sacrificed after 5 h by anesthetic CO_2_ gas exposure.

Blood was collected from an inferior vena cava using a heparinated syringe, and plasma was separated by centrifugation and stored at −80 °C. The left lobes of liver were immediately collected and either fixed in 10% formalin for histology or snap-frozen in liquid nitrogen and stored at −80 °C for biochemical analysis.

### 2.5. Plasma Biochemical Assays

Plasma alanine aminotransferase (ALT) and aspartate aminotransferase (AST) levels were measured using kits from IVD Lab (Uiwang, Korea) and a spectrophotometer (Shimadzu UV-1800 spectrophotometer, Tokyo, Japan). The ALT and AST kits have the following features. Sensitivity is a blank measure of optical density (OD) values over 1.0000; accuracy is within 100 ± 10% when measuring control serum; reproducibility is within 5% of coefficient of variation (CV) when repeated 10 times; linearity is 1000 IU/l. Plasma TNF-α and IL-6 levels were determined using enzyme-linked immunosorbent assay (ELISA) kits (#88-7324-88 and #88-7064-88, respectively) from Invitrogen (Waltham, MA, USA) according to the manufacturer’s instruction. The TNF-α kit has a sensitivity of 8 pg/mL, an assay range of 8–1000 pg/mL, and interassay CV and intraassay CV of less than 10%. The IL-6 kit has a sensitivity of 4 pg/mL, an assay range of 4–500 pg/mL, and interassay CV and intraassay CV of less than 10%.

### 2.6. Cell Culture and Treatment

Raw264.7 cells were obtained from the Americal Type Culture Collection (ATCC, Rockwille, MD, USA) and maintained in Dulbecco’s modified Eagle medium (DMEM), supplemented with 10% fetal bovine serum and 1% penicillin/streptomycin (Thermo Fisher Scientific, Waltham, MA, USA). The cells were incubated at 37 °C in a humidified chamber containing 5% CO_2_ and 95% air.

Lipopolysaccharides (LPS) and specific inhibitors, SB203580 (p38 inhibitor), SP600125 (JNK inhibitor), PD98059 (ERK inhibitor), and LY294001 (PI3K inhibitor), were purchased from Sigma-Aldrich (St. Louis, MO, USA). LPS and the inhibitors were pretreated at 1 h prior to vehicle (water) or PG extracts at concentrations as indicated in figure legends.

### 2.7. Cell Viability

Cell viability was determined by 3-(4,5-dimethyldiazol-2-yl)-2,5-diphenyltetrazolium bromide (MTT) assay, as previously described [22]. Briefly, after drug treatment, 200 μL of MTT solution were added to each well (final 0.1 mg/mL) and cells were incubated for 4 h at 37 °C. The supernatant was aspirated, and formazan crystals were dissolved in 200 μL dimethyl sulfoxide. Absorbance at 570 nm was measured using an Infinite 200 microplate reader (Tecan Austria GmbH, Grödig, Austria).

### 2.8. Extracellular NO measurement

As previously described, extracellular nitric oxide (NO) concentrations were determined [23,24]. After drug treatment, 100 μL of the culture medium were mixed with an equal volume of Griess reagent (0.2% N-1-naphthylethylenediamine dihydrochloride and 2% sulfanilamide in 5% phosphoric acid). Absorbance at 550 nm was measured using an Infinite 200 microplate reader (Tecan), and the nitrite concentration was determined using sodium nitrite standards.

### 2.9. Histology and Terminal Deoxynucleotidyl Transferase dUTP Nick End Labeling (TUNEL) Assay

The fixed livers were processed for paraffin embedding and sectioned to 5-μm thickness. Sections were stained with Hematoxylin and Eosin (H&E) (Sigma-Aldrich, St. Louis, MO, USA) by a standard protocol.

For 4-Hydroxynonenal (4-HNE) immunehistochemical staining, sections were boiled in citric acid buffer (10 mM, pH 6.0) for 20 min for antigen retrieval. After blocking in 10% normal horse serum, sections were incubated with 4-HNE antibody (ab48506, Abcam) overnight at 4 °C. Sections were then incubated with a biotinylated secondary antibody (BP-9200, Vector Laboratories, Burlingame, CA, USA) for 1 h at room temperature. Sections were washed and incubated in avidin–biotin–peroxidase complex solution (ABC solution; Vector Laboratories) and developed using a 3,3ʹ-diaminobenzidine (DAB) Peroxidase Substrate Kit (Vector Laboratories). TUNEL assay was performed using an in-situ cell death detection kit (Roche Molecular Biochemicals, Mannheim, Germany) according to the manufacturer’s protocol. All images were captured using a CKX41 light microscope (Olympus, Tokyo, Japan).

### 2.10. Western Blot Analysis

Liver tissues or Raw264.7 cells were homogenized in ice-cold radioimmunoprecipitation assay (RIPA) buffer with protease inhibitors (Thermo Fisher Scientific), sonicated, and incubated for 20 min on ice. After centrifugation, the supernatant was transferred to a clean tube and protein concentration was determined using a Bio-Rad protein assay kit (Bio-Rad, Hercules, CA, USA). The proteins were separated by sodium dodecyl sulphate-polyacrylamide gel electrophoresis (SDS-PAGE) and transferred to polyvinylidene difluoride (PVDF) membranes. After blocking, the membranes were incubated with primary antibodies at 4 °C overnight. The uncleaved caspase-3 (cs9662), cleaved caspase-3 (cs9661), uncleaved caspase-8 (cs9746), cleaved caspase-8 (cs8592), and PARP-1 (cs9542), IκBα (cs9242), p38 (cs9212), p-p38 (cs9211), ERK (cs4696), p-ERK (cs4377), AKT (cs9272), p-AKT (cs9271), JNK(cs9252), and p-JNK (cs9251) antibodies were from Cell Signaling Technology (Danvers, MA, USA). The iNOS (sc-650) antibody was from Santa Cruz (Dallas, TX, USA), the 4-HNE antibody (ab48506) was from abcam (Cambridge, MA, USA), and the β-actin antibody was from Sigma-Aldrich. Then, the membranes were incubated with horseradish–peroxidase-conjugated secondary antibodies (Bio-Rad) at room temperature for 1 h and protein bands were detected with enhanced chemiluminescence (ECL) substrates (Bio-Rad). The ChemiDoc XRS+ System (Bio-Rad) was used to analyze the band density.

### 2.11. Reverse Transcription and Quantitative Polymerase Chain Reaction (qPCR)

Total RNA was extracted using Trizol (Thermo Fisher Scientific) and converted into cDNA with the RevertAid Reverse Transcription System (Thermo Fisher Scientific) according to the manufacturer’s protocol. Quantitative PCR was performed on the CFX Connect Real-Time PCR System by using iQ SYBR Green Supermix (Bio-Rad). Relative mRNA levels were normalized to those of GAPDH. The primer sequences are as (forward, f; reverse, r) follows: TNF-α(f) 5′-CATATACCTGGGAGGAGTCT-3′; TNF-α(r) 5′-GAGCAATGA CTCCAAAGTAG-3′; IL-6(f) 5′-CCAATTCATCTTGAAATCAC-3′; IL-6(r) 5′-GGAATGTCCACAAACTGATA-3′; MCP-1(f) 5′-ACCTTTGAATGTGAAGTTGA-3′, MCP-1(r) 5′-CTACAGAAGTGCTTGAGGTG-3′; MIP-2(f) 5′-AGAGGGTGAGTTGGGAACTA-3′; and MIP-2(r) 5′-GCCATCCGACTGCATCTATT-3′.

### 2.12. Statistical Analysis

Statistical difference was determined by using one-way analysis of variance (ANOVA), followed by Tukey’s post-hoc analysis (GraphPad Prism 7 Software, v.7.00, La Jolla, CA, USA). Data are presented as the mean ± standard error of the means (SEM) and a *p* value < 0.05 was considered statistically significant.

## 3. Results

### 3.1. BPG and FBPG Extracts are Rich in Phenolic Acid, Flavonoid, and Triterpenoide Contents

We determined the contents of phenolic acid, flavonoid, and triterpenoide in PG extracts by HPLC analysis. First, among the nine phenolic acids, the contents of protocatechuic acid, chlorgenic acid, and p-hydrobenzoic were significantly increased in BPG and FBPG, and the total phenolic acid content was five times higher than DPG (Table 1). Second, among the eleven flavonoids, the contents of epigallocatechin, catechin, epicatechin, and quercetin were significantly increased in BPG and FBPG, and the total flavonoid content was almost three times higher than DPG (Table 2). Third, triterpenoide contents were also detected significantly in BPG and FBPG (Table 3). The results indicate that extraction and fermentation procedures significantly enhanced the phenolic and flavonoids contents, which may provide pharmacological effects on various diseases. Here, we investigated the anti-inflammatory and hepatoprotective effects on Raw264.7 cells and on endotoxin-induced liver injury in vivo.

### 3.2. BPG and FBPG Extracts Reduces Inflammatory Cytokine and Nitric Oxide in Raw264.7 Cells

**Figure 1:**
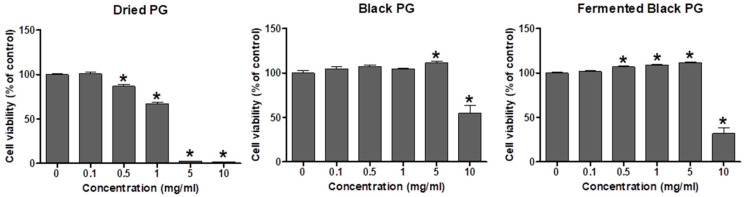
Effect of PG extracts on the viability of Raw264.7 cells. MTT assay was performed to determine cell viability after treatment of PG extracts with 0.1, 0.5, 1, 5, and 10 mg/mL or vehicle for 24 h. The data are presented as the mean ± SEM. * *p* < 0.05 versus vehicle, PG: *Platycodon grandifloras*.

We first determined the highest non-cytotoxic concentration of PG extracts by MTT assay. Raw264.7 cells were treated for 24 h with DPG, BPG, or FBPG at various concentrations, as indicated (Figure 1). DPG began to show cytotoxicity at 0.5 mg/mL, while BPG and FBPG began to show at 10 mg/mL. Interestingly, BPG and FBPG showed a proliferative effect at 5 and 0.5–5 mg/mL, respectively. Thus, we used DPG at 0.1 mg/mL (non-cytotoxic concentration) and BPG and FBPG at 0.1 (same as DPG) and 1 mg/mL (too see any beneficial effects at a higher but non-cytotoxic concentration) for subsequent in vitro experiments.

**Figure 2:**
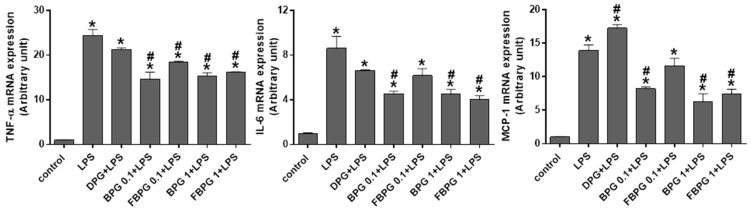
Effect of PG extracts on the expression of pro-inflammatory cytokines in LPS-treated Raw264.7 cells. DPG, BPG, or FBPG were pretreated at 1 h prior to LPS (1 μg/mL), followed by 1-h incubation. DPG was treated at 0.1 mg/mL and other PG extracts at 1 mg/mL. The mRNA expression of TNF-α, IL-6, and MCP-1 was determined by real-time PCR analysis. Relative mRNA levels were normalized to those of GAPDH. The data are presented as the mean ± SEM. * *p* < 0.05 versus vehicle. ^#^ *p* < 0.05 versus LPS, TNF-α: tumor necrosis factor, IL-6: interleukin-6, MCP-1: Monocyte chemoattractant protein-1, LPS: lipopolysaccharide, DPG: dried *Platycodon grandifloras*, BPG: Black *Platycodon grandifloras*, FBPG: fermented black *Platycodon grandifloras*.

**Figure 3:**
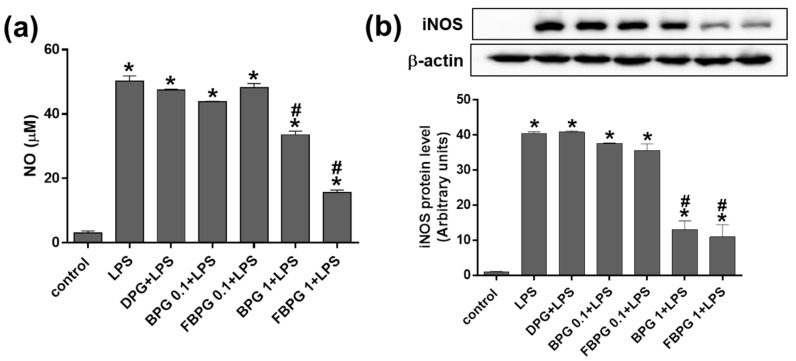
BPG and FBPG extracts reduce the NO release and iNOS expression in LPS-treated Raw264.7 cells. DPG, BPG, or FBPG were pretreated at 1 h prior to LPS (1 μg/mL). DPG was treated at 0.1 mg/mL and other PG extracts at 0.1 and 1 mg/mL. (**a**) After 24 h, medium was collected and mixed with Griess reagent for extracellular NO measurement. (**b**) After 8 h, cell lysates were subjected to Western blotting to analyze the iNOS expression. Relative protein levels were normalized to those of β-actin; the quantification is included. The data are presented as the mean ± SEM. * *p* < 0.05 versus vehicle. ^#^ *p* < 0.05 versus LPS. NO: nitric oxide, iNOS: inducible NO synthesis.

Next, we investigated whether PG extracts have an anti-inflammatory effect on lipopolysaccharide (LPS)-treated Raw264.7 cells. Cells were pretreated with DPG, BPG, or FBPG at 1 h prior to LPS (1 μg/mL) treatment, followed by 1-h incubation. The mRNA expression of tumor necrosis factor (TNF)-α, interleukin 6 (IL-6), and monocyte chemoattractant protein-1 (MCP-1) was measured by qPCR analysis. TNF-α, IL-6, and MCP-1 are inflammatory cytokines and chemokines that are actively transcribed by NF-κB upon LPS stimulation [25,26]. BPG and FBPG reduced significantly all three proinflammatory cytokines induced by LPS (Figure 2). To further elucidate the effect of PG extracts on nitric oxide (NO) levels, we measured extracellular NO concentration and intracellular expression of inducible NO synthesis (iNOS). Cells were pretreated with DPG, BPG, or FBPG at 1 h prior to LPS (1 μg/mL) treatment. After 24-h incubation with LPS, extracellular NO levels were measured (Figure 3a), and after 8-h incubation with LPS, iNOS protein levels were determined by Western blot analysis (Figure 3b). LPS increased significantly the extracellular NO and iNOS protein levels, which were downregulated by BPG and FBPG at 1 mg/mL concentration. These results suggest that the DPG cytotoxicity was alleviated during extraction and fermentation procedures, and FBPG were particularly effective on reducing NO, a critical inflammatory mediator induced by LPS in Raw264.7 cells.

### 3.3. BPG and FBPG Extracts Reduces iNOS Expression through p38 and ERK Signaling Pathway in Raw264.7 Cells

**Figure 4:**
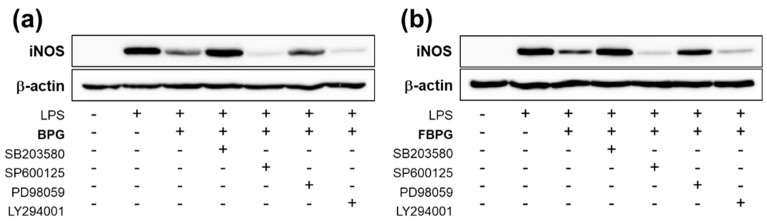
BPG and FBPG extracts reduces the iNOS expression through p38 and ERK signaling in LPS-treated Raw264.7 cells. Specific inhibitors of p38 (SB203580), JNK (SP600125), ERK (PD98059), and PI3K (LY294001) were pretreated at 1 h prior to BPG (**a**) or FBPG (**b**) at 1 mg/mL concentration. After 8-h incubation with LPS (1 mg/mL), cell lysates were subjected for Western blotting to analyze the iNOS expression. Relative protein levels were normalized to those of β-actin.

To investigate which signal pathways are involved in BPG and FBPG effects on iNOS reduction in LPS-induced Raw264.7 cells, specific inhibitors of p38, c-Jun N-terminal kinase (JNK), extracellular signal-regulated kinase (ERK), and phosphoinositide 3-kinase (PI3K) were used to block the action of specific kinases. Cells were pretreated with SB203580 (p38 inhibitor), SP600125 (JNK inhibitor), PD98059 (ERK inhibitor), or LY294001 (PI3K inhibitor) at 1 h prior to BPG or FBPG treatment. After 8-h incubation with LPS (1 mg/mL), iNOS protein levels were determined by Western blot analysis (Figure 4). The results show that iNOS levels induced by LPS were decreased by BPG and FBPG treatment; however, these were blocked by SB203580 and PD98059, indicating that p38 and ERK signaling pathway was involved.

**Figure 5:**
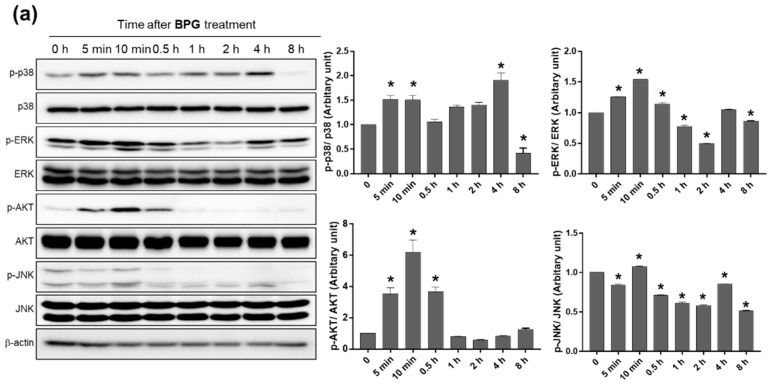

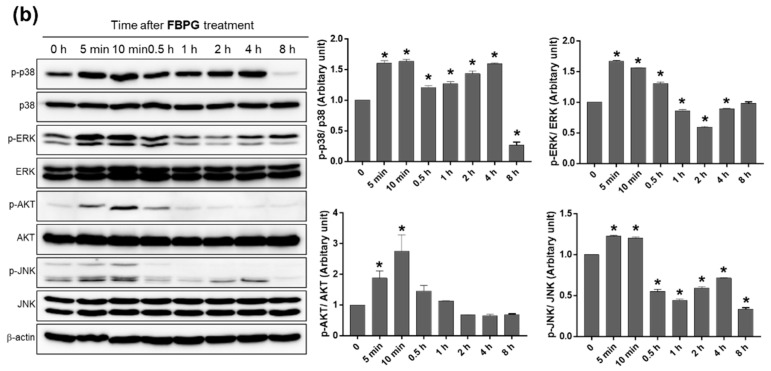
The effect of BPG and FBPG extracts on the activation of p38, ERK, AKT, and JNK signaling kinases in Raw264.7 cells. BPG (**a**) or FBPG (**b**) were treated at 1 mg/mL concentration for various times (5 and 10 min and 0.5, 1, 2, 4, and 8 h). Cell lysates were subjected for Western blotting to analyze the phosphorylation of p38, ERK, AKT, and JNK. Phosphorylated protein levels were normalized to respective total protein levels. * *p* < 0.05 versus control.

To characterize the signaling pathway associated with BPG and FBPG treatment in Raw264.7 cells, the phosphorylation levels of p38, ERK, AKT, and JNK were determined at 5 and 10 min and 0.5, 1, 2, 4, and 8 h after BPG or FBPG treatment. Phosphorylation levels started increasing at 5 min after treatment and decreased during 0.5–1 h, except p38, which lasted up to 4 h (Figure 5); these changing patterns were similar in cells treated with BPG or FBPG. The results might provide molecular insights to understand the effects of BPG and FBPG on stressed or diseased cells where these signaling pathways have been significantly altered.

### 3.4. FBPG Extracts Reduces Hepatic Damage and Necrosis in an LPS/GalN-Induced Liver Injury

**Figure 6:**
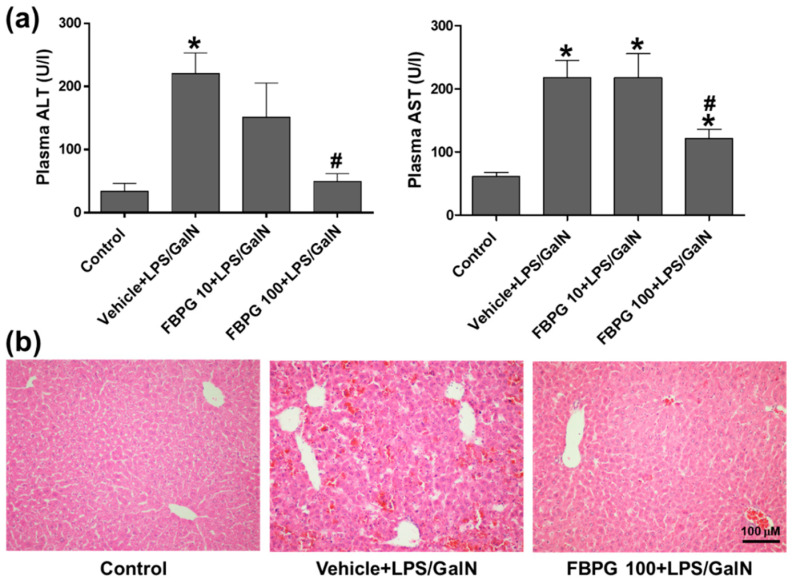
FBPG extract decreases plasma ALT and AST and attenuates liver damage in LPS/GalN-injected mice. C57BL/6 mice were administered orally with vehicle or FBPG (10 or 100 mg/kg) at 1 h prior to LPS/GalN injection (n = 8 each). The mice were sacrificed after 5 h and sham mice (n = 5) were included as control. (**a**) Blood was collected, and plasma ALT and AST levels were measured. (**b**) Liver sections were processed for H&E staining and representative images are shown. The data are presented as the mean ± SEM. * *p* < 0.05 versus control. ^#^ *p* < 0.05 versus vehicle + LPS/GalN. Scale bar, 100 μm. GalN: galactosamine, ALT: alanine aminotransferase, AST: aspartate aminotransferase.

To investigate the effect of FBPG on an endotoxin-induced acute liver injury, mice were orally administered with vehicle or FBPG (10 or 100 mg/kg) at 1 h prior to an LPS/GalN (10 μg/kg and 700 mg/kg, respectively) injection. Liver injury was assessed by measuring plasma ALT and AST levels and liver histological analysis. FBPG treatment (100 mg/kg) reduced ALT and AST levels significantly (Figure 6a). Consistently, the hepatic structural disturbance, hemorrhage, inflammatory cell infiltration, and hepatocellular necrosis were attenuated by FBPG treatment in LPS/GalN-injected mice compared with sham mice (Figure 6b).

### 3.5. FBPG Extract Reduces Hepatic Inflammation, Apoptosis, and Lipid Peroxidation in an LPS/GalN-Induced Liver Injury

**Figure 7:**
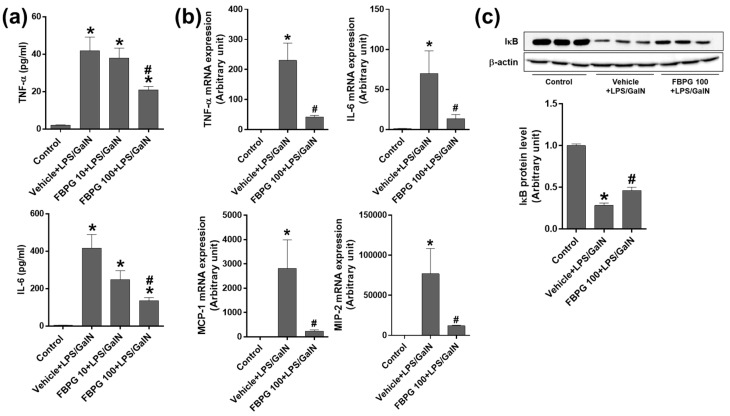
FBPG extract decreases pro-inflammatory cytokines and restores the hepatic IκBα expression in LPS/GalN-injected mice. C57BL/6 mice were administered orally with vehicle or FBPG (10 or 100 mg/kg) at 1 h prior to LPS/GalN injection (*n* = 8 each). The mice were sacrificed after 5 h and sham mice (*n* = 5) were included as control. (**a**) Blood was collected, and plasma TNFα and IL-6 levels were measured. (**b**) The mRNA expression of TNF-α, IL-6, MCP-1, and MIP-2 was determined by real-time PCR analysis. Relative mRNA levels were normalized to those of GAPDH. (**c**) Hepatic tissue lysates were subjected to Western blotting to analyze the IκBα expression. Relative protein levels were normalized to those of β-actin; the quantification is included. The data are presented as the mean ± SEM. * *p* < 0.05 versus control, and ^#^ *p* < 0.05 versus vehicle + LPS/GalN.

To determine the effect of FBPG on an endotoxin-induced hepatic inflammation, we analyzed the plasma TNFα and IL-6 levels (Figure 7a), and hepatic mRNA levels of pro-inflammatory cytokines, TNF-α, IL-6, MCP-1, and macrophage inflammatory protein 2 (MIP-2) (Figure 7b). LPS/GalN-injected mice exhibited a significant increase in the plasma and hepatic cytokine levels, which were significantly reduced by FBPG treatment. In addition, we analyzed the expression of IκBα, which inhibits the NF-κB transcriptional factor. Consistently, FBPG alleviated the degree of IκBα degradation induced by LPS/GalN (Figure 7c).

**Figure 8:**
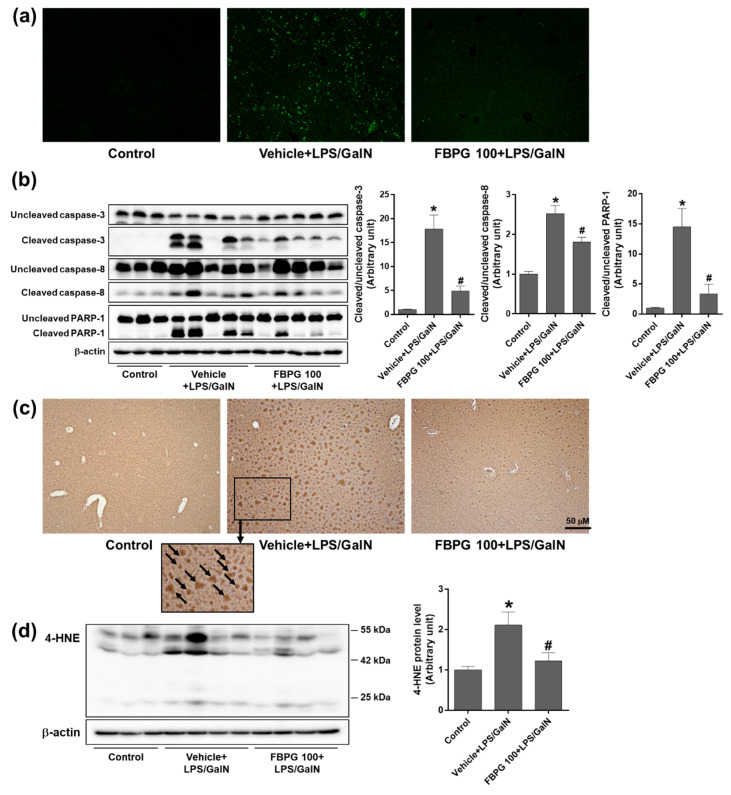
FBPG extract reduces hepatic apoptosis and lipid peroxidation in LPS/GalN-injected mice. C57BL/6 mice were administered orally with vehicle or FBPG (10 or 100 mg/kg) at 1 h prior to LPS/GalN injection (*n* = 8 each). The mice were sacrificed after 5 h and sham mice (*n* = 5) were included as control. (**a**) Liver sections were processed for TUNEL staining and representative images are shown. (**b**) Hepatic tissue lysates were subjected to Western blotting to analyze the expression of cleaved caspase-3, caspase-8, and PARP-1. Relative protein levels were normalized to those of uncleaved forms or β-actin; the quantification is included. (**c**) Liver sections were processed for 4-HNE immunohistochemical staining and representative images are shown. (**d**) Hepatic tissue lysates were subjected to Western blotting to analyze the 4-HNE expression. Relative protein levels were normalized to those of β-actin; the quantification is included. The data are presented as the mean ± SEM. * *p* < 0.05 versus control. ^#^ *p* < 0.05 versus vehicle + LPS/GalN. Scale bar, 50 μm.

Next, we investigated whether FBPG protects from hepatic apoptosis. Compared to sham, LPS/GalN-injected mice showed a dramatic increase in TUNEL-positive cells, which was reduced dramatically by FBPG treatment (Figure 8a). Consistently, hepatic levels of cleaved caspase-3, caspase-8, and poly(ADP-ribose) polymerase (PARP)-1 were attenuated significantly by FBPG treatment compared to vehicle in LPS/GalN-injected mice (Figure 8b).

To determine an antioxidant effect of FBPG, oxidative stress was assessed by 4-HNE, a marker of lipid peroxidation. The immunohistochemical staining showed that the 4-hydroxynonenal (HNE) levels increased significantly in LPS/GalN-injected mice compared with sham, which decreased upon FBPG treatment (Figure 8c). Consistently, the Western blotting showed that hepatic expression of 4-HNE was decreased by FBPG treatment in LPS/GalN-injected mice (Figure 8d). These results indicate that FBPG extract exerts anti-inflammatory, anti-apoptotic, and antioxidant effects, attenuating endotoxin-induced hepatic injury.

## 4. Discussion

The present study investigated the effects of PG extract, acting as an anti-inflammatory and hepatoprotective agent. Raw PG was processed by an optimized extraction and fermentation method to become BPG and FBPG. HPLC analysis showed that phenolic acids, flavonoids, and triterpenoides were rich in BPG and FBPG extracts. The results show that BPG and FBPG treatment reduced inflammatory cytokine and nitric oxide levels in Raw264.7 cells; reduction of iNOS expression, was particularly through p38 and ERK signaling pathway. FBPG treatment clearly showed hepatoprotective effects in LPS/GalN-injected mice by significantly reducing the plasma ALT and AST levels and hepatic necrosis. The treatment also reduced the hepatic inflammatory response and markers of apoptosis and oxidative stress induced by LPS/GalN, which strongly suggests its therapeutic potential for acute liver injury.

PG is known as doraji (Korean), jiegeng (Chinese), kikyo (Japanese), and balloon flower (English) and has a long history as a food and herbal medicine in Asia. Studies have shown that the roots of PG contain a group of various biologically active components, the main active compound being triterpene saponins, especially platycosides [15]. Our study also showed that BPG and FBPG extracts contain platycodin D3, deapioplatycodin D, and polygalcin D. Platycodon D exhibits a strong antioxidant activity against peroxyl radicals, comparable to the glutathione in the Total Oxidant-Scavenging Capacity (TOSC) assays [27].

During extraction and fermentation processes, the content of phenolic acids and flavonoids in BPG and FBPG is enhanced, resulting in the antioxidant and anti-inflammatory properties of the extracts. Chlorgenic acid, p-hydrobenzoic acid, and protocatechuic acid were enhanced in the extracts; these are major metabolites of antioxidant polyphenols or phenolic derivatives found in coffee, coconut fruit, mushroom, green tea, and more [28,29]. Epigallocatechin, epicatechin, and catechin are flavonoids found in green tea, berries, nuts, cocoa, and pome fruits, and have antioxidant, anti-inflammatory, and cardiovascular functions [30]. Thus, these polyphenols and flavonoids, derived from natural medicinal plants, are considered important resources for human health and diseases.

PG is known for its pharmacological effects on the respiratory system, especially cough, phlegm, and asthma. Particularly, platycosides have shown to stimulate mucus secretion and mucin release, and reduce airway inflammation and allergic reactions [31,32]. PG has been widely used to treat many chronic inflammatory diseases such as arthritis and chronic bronchitis. PG has been shown to suppress inflammatory cytokine and prostaglandin E2 production, iNOS and cyclooxygenase-2 expression through blocking NF-κB activation in LPS-induced A549 airway epithelial cells, Raw264.7 macrophages, and BV2 microglial cells [33,34,35]. Consistently, our study showed that BPG and FBPG extracts reduced pro-inflammatory cytokines, NO release, iNOS expression, and IκBα degradation.

Immune cells initiate an inflammatory response through the tremendous production of pro-inflammatory cytokines and mediators involved in various kinase signaling cascades for NF-κB activation [25]. The mitogen-activated protein kinases (MAPKs; p38, ERK1/2, and JNK) and PI3K/AKT signaling pathways are reported as important upstream regulators [36]. LPS has shown to increase phosphorylation of these kinases in Raw264.7 cells, and many medicinal plant extracts have been shown to mitigate these kinase signaling and NF-kB activation through antioxidant properties, such as induction of nuclear factor erythroid 2–related factor 2 (Nrf2) and heme oxygenase (HO-1) and inhibition of iNOS and cyclooxygenase-2 [37,38,39]. In this study, for the first time, we confirmed that BPG and FBPG extracts exert a beneficial effect by reducing iNOS expression, especially through p38 and ERK signaling pathways in LPS-induced Raw264.7 cells. Interestingly, we found that the phosphorylation of p38, ERK, AKT, and JNK was immediately increased within 5–10 min after treatment with BPG and FBPG extracts, and decreased by 8 h except for p38, indicating that the extracts have anti-inflammatory effects by blocking these kinases in LPS-treated Raw264.7 cells.

Oxidative stress and inflammation are closely linked to the pathogenesis of acute and chronic liver diseases, where the endogenous antioxidant systems are frequently impaired, leading to severe tissue damages [40,41]. This suggests that antioxidants have effective therapeutic potentials for those patients. Previously, PG extracts or platycosides have been shown to alleviate liver damages by increasing antioxidant potential in acute ethanol, carbon tetrachloride, or tert-butyl hydroperoxide-induced hepatotoxicity in mice [42,43,44]. Our results also show a significant reduction in lipid peroxidation by FBPG treatment in LPS/GalN-injected mice. Correlatively, HPLC analysis showed that BPG and FBPG extracts were enriched in platycosides, phenolic, and flavonoid compounds.

Interestingly, the synergistic effect of quercetin and catechin (or docosahexaenoic acid) has been reported by reducing pro-inflammatory cytokines and NF-kB activation via inhibiting MAPK signaling in LPS-treated Raw264.7 cells [45,46]. The combination of quercetin and galangin has also shown improved anti-inflammatory effects in the mouse model of atopic dermatitis [47]. This suggests that catechins, quercetin, and other antioxidants rich in BPG and FBPG may have synergistic effects on reducing inflammation and oxidative stress by downregulating the MAPK and ERK signaling pathways. Further research is needed on how the extracts regulate the downstream of these kinases to attenuate LPS-induced inflammation, necrosis, and apoptosis and prevent acute and chronic liver injury.

We performed an MTT assay in vitro to determine the highest non-cytotoxic concentration of the extracts before investigating the therapeutic effect in LPS-treated cells. Interestingly, BPG and FBPG showed no cytotoxicity at up to 5 mg/mL, compared to DPG (Figure 1). As shown in Table 1, Table 2 and Table 3, the contents of phenolic acids, flavonoids, and platycodin D in BPG and FBPG extracts, were higher than those in DPG, which contributed to the enhancement of antioxidant potential of BPG and FBPG.

There are no specific toxicity known to laboratory animals unless treated at high doses of plant extracts due to the effects of contaminants and interactions with other drugs and metabolites [48,49]. Without apparent toxicity, 100 mg/kg of FBPG showed significant hepatoprotective effects in LPS/GalN-injected mice in this study. The bioavailability of naturally occurring polyphenols and flavonoids can also be altered through extraction and fermentation processes, by increasing the efficiency of absorption, digestion, and metabolism in the body [29,50].

The general toxicity tests of FBPG was performed by three different ways. First, in the acute toxicity test, 2000 mg/kg of extract were administered orally to eight-week-old SD rats five times at 1-h intervals; there were no death, morbidity, or behavioral abnormality and no changes in body weight, internal organ structure or function. Second, in a dose-range finding study, the No Observed Adverse Effect Level (NOAEL) was 1000 mg/kg for two weeks in SD rats. Third, in the Ames II test, extract up to 1000 mg/kg did not induce a reverse mutation of *Salmonella Typhimurium (His-)*. Consistent with the general toxicity tests of FBPG, platycodin D did not show any pathological changes up to 2000 mg/kg administered to mice [51]. A major limitation of the study is that the extracts were prepared using a standardized protocol developed by the lab of Kye Man Cho, but the reproducibility has not yet been confirmed.

In conclusion, FBPG plays anti-inflammatory, antioxidant, and anti-apoptotic roles, alleviating endotoxin-induced acute liver injury, and it may further prevent other complications of the brain, kidney, or vascular system associated with ALF. Processing raw PG into FBPG extract may be clinically useful by improving the pharmacologically active ingredients at lower doses.

## Figures and Tables

**Table 1:** Phenolic acids contents.

Contents ^1^ (mg/g d.w.)	DPG	BPG	FBPG
Gallic acid	6.10	ND	ND
Protocatechuic acid	11.57	133.57	126.71
Chlorgenic acid	38.26	235.66	244.51
p-Hydrobenzoic acid	14.80	61.25	57.72
Vanillic acid	ND	11.10	12.44
p-Coumaric acid	1.09	ND	ND
Ferulic acid	5.88	ND	ND
Vertaric acid	9.26	ND	17.10
t-Cinnamic acid	0.73	0.79	0.86
Total	87.69	442.37	459.34

^1^ All values are presented as the mean from three independent determination. ND, non-detectable, DPG: dried *Platycodon grandifloras*, BPG: Black *Platycodon grandifloras*, FBPG: fermented black *Platycodon grandifloras*.

**Table 2:** Flavonoids contents.

Contents ^1^ (mg/g d.w.)	DPG	BPG	FBPG
Epigallocatechin	160.67	323.83	397.05
Catechin	15.22	57.84	43.23
Epicatechin	20.90	130.39	146.25
Epigallocatechin gallate	5.39	58.24	ND
Vanillin	ND	1.23	ND
Rutin	9.86	ND	ND
Catechin gallate	8.23	ND	ND
Quercetin	130.32	320.11	345.23
Naringin	ND	25.14	29.24
Naringenin	8.92	14.86	6.61
Formonoetin	3.82	3.45	3.61
Total	363.33	935.09	971.22

^1^ All values are presented as the mean from three independent determination. ND, non-detectable.

**Table 3:** Triterpenoide contents.

Contents ^1^ (mg/g d.w.)	DPG	BPG	FBPG
Platycodin D3	ND	1.67	0.87
Deapioplatycodin D	0.18	0.77	0.50
Polygalcin D	ND	0.27	0.19
Total	0.18	2.71	1.56

^1^ All values are presented as the mean from three independent determination. ND, non-detectable
